# Pupillometry reveals the physiological underpinnings of the aversion to holes

**DOI:** 10.7717/peerj.4185

**Published:** 2018-01-04

**Authors:** Vladislav Ayzenberg, Meghan R. Hickey, Stella F. Lourenco

**Affiliations:** Department of Psychology, Emory University, Atlanta, GA, United States of America

**Keywords:** Holes, Vision, Fear, Pupillometry, Trypophobia, Psychophysiology, Disgust

## Abstract

An unusual, but common, aversion to images with clusters of holes is known as trypophobia. Recent research suggests that trypophobic reactions are caused by visual spectral properties also present in aversive images of evolutionary threatening animals (e.g., snakes and spiders). However, despite similar spectral properties, it remains unknown whether there is a shared emotional response to holes and threatening animals. Whereas snakes and spiders are known to elicit a fear reaction, associated with the sympathetic nervous system, anecdotal reports from self-described trypophobes suggest reactions more consistent with disgust, which is associated with activation of the parasympathetic nervous system. Here we used pupillometry in a novel attempt to uncover the distinct emotional response associated with a trypophobic response to holes. Across two experiments, images of holes elicited greater constriction compared to images of threatening animals and neutral images. Moreover, this effect held when controlling for level of arousal and accounting for the pupil grating response. This pattern of pupillary response is consistent with involvement of the parasympathetic nervous system and suggests a disgust, not a fear, response to images of holes. Although general aversion may be rooted in shared visual-spectral properties, we propose that the specific emotion is determined by cognitive appraisal of the distinct image content.

## Introduction

The relation between fear and disgust has long been debated (Darwin, 1872/1998; Rachman, 1998; Rozin & Fallon, 1987; Woody & Teachman, 2000). Although both emotions elicit defensive responses and have clear adaptive value to the organism, accumulating evidence suggests that fear and disgust are accompanied by distinct behavioral profiles and physiological underpinnings (Calder, Lawrence & Young, 2001; Chapman & Anderson, 2012; Öhman et al., 2007; Phillips et al., 1998). Fear is considered a response to perceived danger (Rachman, 1998), with activation from the sympathetic nervous system (Bradley et al., 2008; Gray, 1987; Kreibig, 2010). By contrast, disgust is considered a reaction to contamination (Davey, Forster & Mayhew, 1993; Rozin & Fallon, 1987) and is commonly associated with activation of the parasympathetic nervous system (Calder, Lawrence & Young, 2001; Murphy, Nimmo-Smith & Lawrence, 2003). When experienced in excess, both fear and disgust can become associated with phobic reactions. In particular, animal phobias such as arachnophobia and ophidiophobia are thought to be predominately rooted in fear (Öhman & Mineka, 2001; Sarlo et al., 2002), whereas blood-injection-injury phobia and obsessive-compulsive disorder (OCD) are more strongly associated with disgust (Hermann et al., 2007; Tolin et al., 1997). In the present study, we examined individuals’ reactions to images known to elicit trypophobia, an unusual, but common, phobia with ambiguous emotional underpinnings (Cole & Wilkins, 2013; Le, Cole & Wilkins, 2015). And as discussed in more detail below, we used pupillometry in a novel attempt to dissociate fear and disgust in the case of the trypophobic response.

Trypophobia has been described as an aversion to a cluster of innocuous holes (Aminuddin & Lotfi, 2017; Cole & Wilkins, 2013; Imaizumi et al., 2016a). Though not listed in the Diagnostic and Statistical Manual of Mental Disorders (DSM-5; American Psychiatric Association, 2013), recent empirical studies and Internet-based support groups confirm the specificity of the aversion to images in both self-described trypophobes and the general population depicting holes or porous textures (e.g., lotus seed plant, honeycomb, and aerated chocolate). An analysis of the visual spectral properties of such images revealed an excess of contrast energy in midrange spatial frequencies, suggesting a perceptual basis to this aversion (Cole & Wilkins, 2013; Le, Cole & Wilkins, 2015; Sasaki et al., 2017). Consistent with such an account, Van Strien & Van der Peijl (2015) found that early posterior negativity (EPN), an ERP component thought to reflect visual processing of emotionally significant stimuli, was larger for trypophobic images than non-trypophobic images (i.e., birds) in occipital cortex.

Although an account emphasizing the visual-perceptual roots of trypophobia has gained increasing empirical support, many questions remain about the nature of the emotional response in trypophobia as well as its physiological etiology. Trypophobia is often referred to as a “fear” of holes (Cole & Wilkins, 2013; Le, Cole & Wilkins, 2015; Pipitone, Gallegos & Walters, 2017) and, indeed, Cole & Wilkins (2013) demonstrated that the spectral properties that characterized trypophobic images (i.e., high contrast energy at midrange spatial frequencies) were comparable to those in images of evolutionary threatening animals such as snakes and spiders, which are known to induce fear reactions in individuals (Öhman, Flykt & Esteves, 2001; Sarlo et al., 2002; Vagnoni, Lourenco & Longo, 2012). However, testimonials of self-described trypophobes suggest another possibility–namely, that the emotional reaction may, instead, reflect disgust. When describing their aversion to holes, individuals use terms such as “repulsive” and “gross” (Cole & Wilkins, 2013). Empirical support for disgust as the core emotion in trypophobic reactions comes from a research study by Imaizumi et al. (2016a) in which trypophobia proneness (as measured by the Japanese version of the Trypophobia Questionnaire; Imaizumi et al., 2016b) was predicted by disgust (as measured by the Disgust Scale-Revised; Haidt, McCauley & Rozin, 1994; Olatunji et al., 2007). But there were no measures of fear included in this study, making it impossible to rule out that trypophobic reactions reflected fear responses instead of disgust. In summary, questions remain about the accompanying emotional reaction of the aversion to holes. Characterizing the emotional etiology of the trypophobic reaction as well as how it may relate to other known phobic reactions (e.g., fear of spiders) will not only inform theoretical models concerned with dissociating fear and disgust (Woody & Teachman, 2000), but it may also play a crucial practical role in evaluating different interventions, since aversive reactions with different emotional underpinnings may, under extreme conditions, necessitate treatments tailored to the specific emotional dysfunction.

In the present study, we used pupillometry to differentiate fear and disgust reactions to images of holes. Pupillometry provides a measure of pupil size, which is regulated by dilator and sphincter muscles (Granholm & Steinhauer, 2004; Sirois & Brisson, 2014). Crucially, these two muscles are differentially influenced by activity in sympathetic and parasympathetic branches of the nervous system. There is much evidence for a relation between fear and activation of the sympathetic nervous system, known for triggering a “fight-or-flight” response (Bradley et al., 2008; Gray, 1987; Kreibig, 2010). A fear response includes an increase in overall cardiovascular function, namely heart rate acceleration (Folkow, 2000; Levenson, 1988), and a response in the dilator muscles, prompting pupil dilation (i.e., larger pupil size). By contrast, disgust is largely characterized by a response of the parasympathetic nervous system, known for “rest-and-digest” functions (De Jong, Van Overveld & Peters, 2011; Levenson, 1988; Van Overveld, De Jong & Peters, 2009). These functions include slowing of cardiovascular functions, specifically decreased heart rate (Hermann et al., 2007; Kreibig, 2010; Stark et al., 2005), and a response in the sphincter muscles, prompting pupillary constriction (i.e., smaller pupil size).

**Figure 1:**
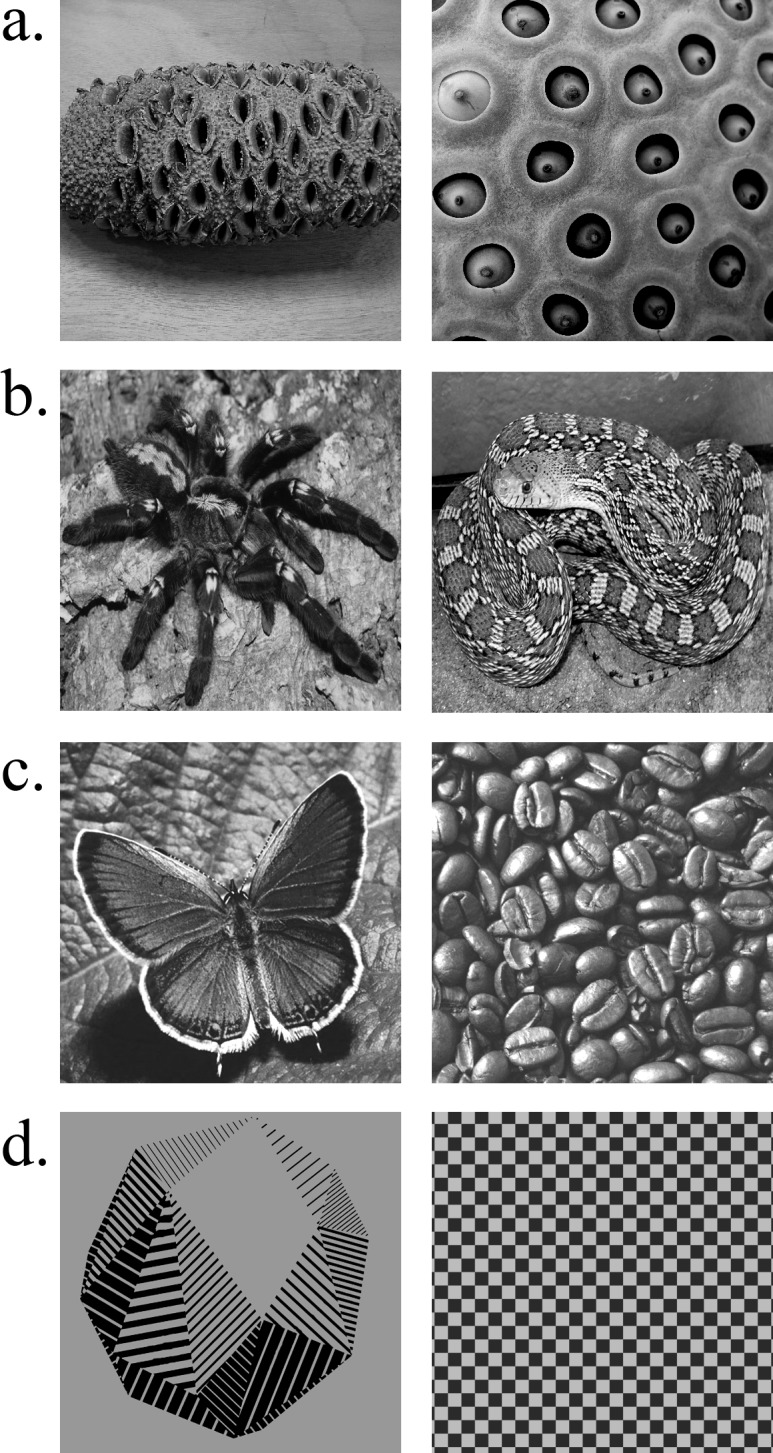
Example stimuli from each stimulus category used in Experiments 1 and 2. (A) Images from the holes category known to elicit an aversive response in trypophobes and the general population. (B) Images from the threat category generally associated with a fear response in individuals with snake and spider phobias (arachnophobia and ophidiophobia, respectively) as well as the general population. (C) Images from the neutral category used as controls in Experiment 1. (D) Images from the control category (i.e., controls for the pupil grating response) included in Experiment 2.

Across two experiments, we compared changes in pupil size when participants viewed images of holes (known to elicit trypophobia; Fig. 1A) and images of threatening animals, namely snakes and spiders (Fig. 1B). Previous research suggests that snakes and spiders largely elicit a fear response (Öhman, Flykt & Esteves, 2001), such that pupil dilation associated with the sympathetic nervous system activation would be expected. If trypophobic images similarly elicit a fear response, then there should be pupil dilation comparable to that for snakes and spiders. However, if images of holes elicit a disgust response, then the prediction is that there will be less dilation (i.e., pupillary constriction), consistent with involvement of the parasympathetic nervous system. As contrastive controls, we also included images of animals and objects not known to elicit a fear or disgust reaction (Fig. 1C; Experiment 1), and non-hole patterns similar in repetitive patterning as the hole images but not associated with trypophobia (Fig. 1D; Experiment 2). Participants’ pupillary responses to hole images were compared to both images of evolutionary threatening animals (i.e., snakes and spiders) and different categories of control images, allowing for comprehensive comparisons and unambiguous interpretations of pupillary responses in these experiments. Notably, images of holes are uncomfortable to individuals in the general population, not simply to those who profess to trypophobia (Cole & Wilkins, 2013; Le, Cole & Wilkins, 2015). We thus tested random samples of participants from a large population of college students in these experiments to inform our understanding of the physiology underlying the general aversion to holes.

## Experiment 1

### Method

#### Participants

Forty-one undergraduate students (*M*_age_ = 19.84 years; 30 females) participated in this experiment for course credit. All participants had normal or corrected-to-normal vision.

#### Stimuli and procedure

Stimulus presentation consisted of 60 images (512 × 512 px; 13.5°× 13.5°): 20 images of “holes” from the trypophobia image set used by Wilkins and colleagues ((Le, Cole & Wilkins, 2015); obtained from Arnold Wilkins), 20 images of snakes and spiders (Vagnoni, Lourenco & Longo, 2012), and 20 neutral objects (see Fig. 1). The trypophobia image set included individual objects with multiple holes (e.g., sea sponge) as well as images of porous textures (e.g., lotus seed plant; see Fig. 1A). Images of snakes and spiders constituted the “threat” category and were selected so as to include the entirety of the animal (see Fig. 1B). The neutral category of images included individual objects (e.g., cup), displays (e.g., a pile of coffee beans), and neutrally-valenced animals (e.g., butterfly; see Fig. 1C). In the case of single objects or animals, the items were presented centrally within the image. All images were centered on a uniform gray background (RGB: 106, 106, 106). Prior to image presentation, participants viewed a gray screen that served as the baseline on each trial. All images were gray-scaled using Adobe Photoshop and equated for luminance using the SHINE toolbox for Matlab (mean luminance = 105.76; Mathworks; Willenbockel et al., 2010), ensuring that any effects of pupil size could not be explained by differences in luminance (Bradley et al., 2008).

Stimulus presentation was controlled with a custom Visual Basic Program and presented on a 22-inch computer monitor (1,920 × 1,080 px; 75 Hz refresh rate). Pupillary responses, specifically pupil area, were recorded using an Eyelink-1000 plus eye-tracker (SR-Research) recording at 1,000 Hz. By default, the eye-tracker records the size of the pupil in arbitrary camera units with a possible resolution as small as 5 µm (0.005 mm; SR-Research). Camera units provide an accurate measure of pupil size across variations in eye shape and camera angle.

During viewing, participants were seated in a chinrest 60 cm from the computer monitor. All participants were tested in the same brightly lit room. Eye gaze was calibrated using a 5-point calibration routine. Each trial began with the baseline phase (6 s) and was followed by the image phase (6 s; see Bradley et al., 2008). Participants were instructed to view the images for the entirety of their presentation. Images were presented in a randomized order.

#### Ethics

Participants provided written informed consent prior to participation. Experimental procedures were approved by the Institutional Review Board (IRB) at Emory University and performed in accordance with IRB guidelines (IRB protocol #003388).

### Results

Following previous research, the interest period for baseline and image phases was set to between 1 and 6 s (Bradley, Sapigao & Lang, 2017). Pupillary responses prior to the first second are thought to include a light reflex and thus were not analyzed (Beatty & Lucero-Wagoner, 2000). To enable comparisons across participants and to account for differences in baseline pupil size, we analyzed the mean percent change in pupil size between baseline and image phases over the full interest period for each phase. The image phase elicits an overall smaller pupil compared to the baseline phase, such that a larger change corresponds to a larger decrease in pupil size. Trials in which participants did not fixate to the screen (during the baseline or image phase) were excluded from the analyses (5.8% of trials).

**Figure 2:**
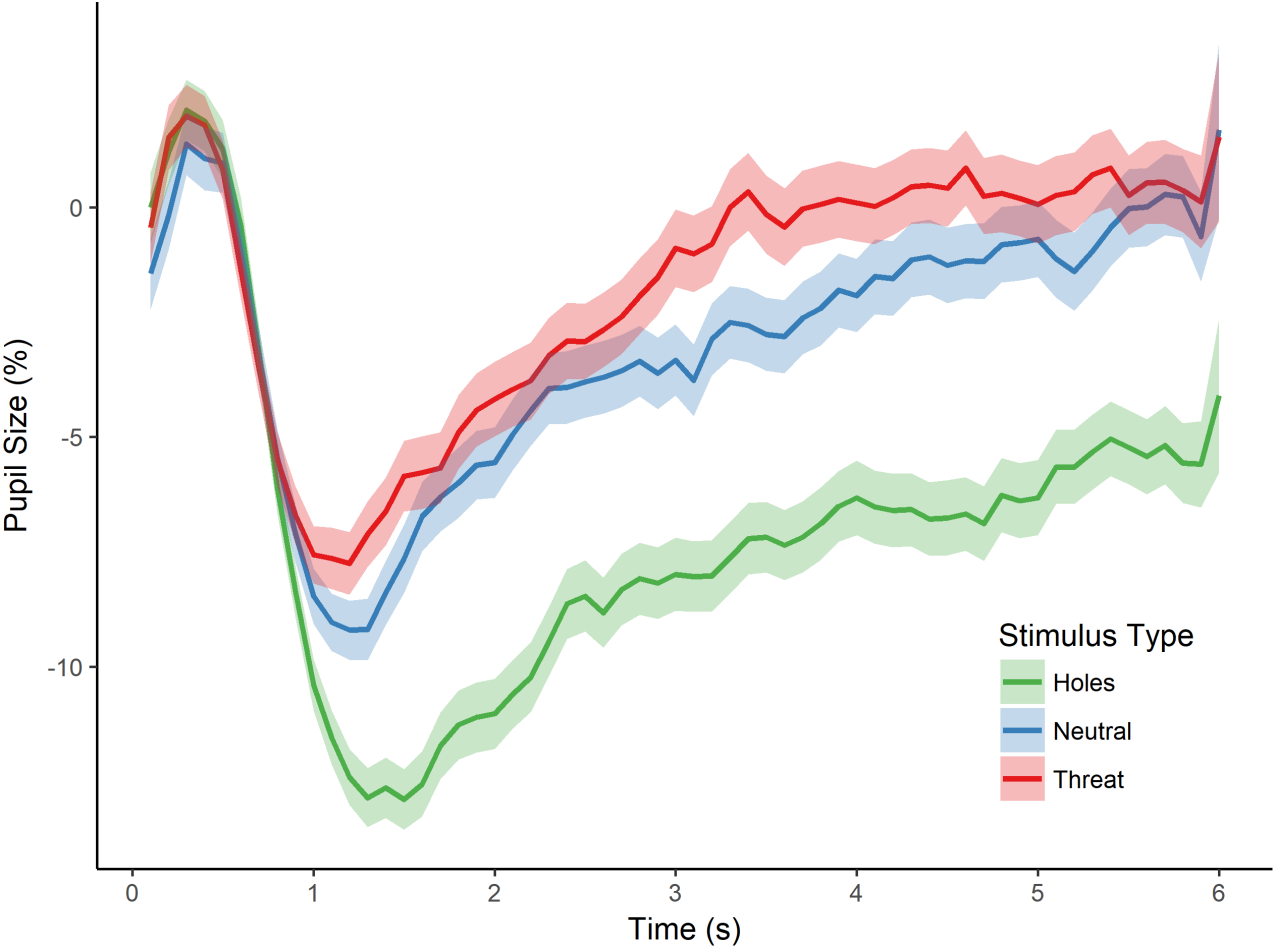
Pupillary waveforms across time for each stimulus type in Experiment 1. The *x*-axis reflects trial time in seconds (s) and the *y*-axis reflects the percentage of pupil-size change from baseline, such that greater percent change corresponds to a smaller pupil size. Shaded colors represent standard error of the mean (SEM).

To test for an effect of stimulus type (i.e., holes, neutral, and threat) on pupillary size, we conducted a repeated measures analysis of variance (ANOVA) with the dependent variable of percent pupil-size change. This analysis revealed a significant main effect of stimulus type, *F*(2, 80) = 18.556, *p* < 0.001, ${\mathrm{\eta }}_{p}^{2}=0.317$ (see Fig. 2). Post-hoc tests, corrected for multiple comparisons using the Holm-Bonferroni method, revealed significantly greater pupil-size change to images of holes than to threatening images (*p* < 0.001), suggesting less dilation and sustained constriction to holes within the interest period, and providing support for a dissociation in pupillary responses between images of holes and threatening animals. Post-hoc tests also revealed a significant difference in pupil-size change between holes and neutral images (*p* < 0.001), such that pupil size was smaller to holes than neutral images, again, consistent with constriction to the images of holes. Taken together, these results suggest an influence of the parasympathetic nervous system during the viewing of holes and are consistent with a disgust response to holes but not the other types of images.

The post-hoc comparisons revealed no significant difference in pupil-size change between neutral images and threatening animals (*p* = 0.879). Although we did not make a specific prediction about the relative pupillary response to threatening images, one might have expected larger pupil size to threatening animals than neutral images because of potential differences in arousal (Bradley et al., 2008). We cannot address this issue directly, however, as we did not collect arousal ratings for any of the images in this first experiment. As discussed below, we control directly for individual differences in arousal level in the next experiment.

### Discussion

The results of this first experiment demonstrate a dissociation between pupil responses when viewing images of holes compared with images of threatening animals (as well as various neutral images). Although photographs (image phase) that follow a blank screen (baseline phase) elicit pupillary constriction for all image categories (Sirois & Brisson, 2014), we found that images of holes resulted in greater pupil-size changes compared to threatening animals (snakes and spiders) and neutral images. These pupil-size changes are consistent with relatively less pupillary dilation throughout the interest period and thus greater constriction for holes. We suggest that participants’ pupillary responses to holes in this experiment lend support to the hypothesis that the trypophobic response experienced by individuals is associated with activation of the parasympathetic nervous system and, thus, more likely associated with the emotional reaction of disgust than fear.

However, because pupillary responses are also heavily influenced by low-level visual properties of an image, independent of its emotional affect, an alternative explanation of our findings is that the differential change in pupil size was due specifically to these properties. Studies examining the relation between pupillary responses and spectral image properties have found that pupil size correlates with contrast frequency gratings, such that high contrast, high spatial frequencies (HSF) are associated with a smaller pupil size, the so-called pupil grating response (Barbur & Thomson, 1987; Cocker et al., 1994). Thus, it is possible that the sustained pupillary constriction to holes observed in Experiment 1 was not reflective of a disgust response but, instead, a response to high contrast, HSF information in these images. To address this possibility, in a subsequent experiment, we included a novel set of neutral images that contained high-contrast repeating patterns similar to those in images of holes (e.g., checkboard; see Fig. 1D). Crucially, unlike the images of holes, these images are not known to elicit trypophobia.

Yet another possibility not addressed in Experiment 1 was that arousal level affected the pupillary response. Bradley et al. (2008) found a relation between arousal and pupillary response, such that greater arousal elicited greater dilation. Indeed, hole images may have been less arousing than the threatening category and, therefore, less likely to cause dilation. In the subsequent experiment, we addressed this possibility by collecting arousal ratings for each image and including participants’ arousal ratings in our analyses of pupillary responses to the presented images.

## Experiment 2

### Method

#### Participants

Forty-four undergraduate students (*M*_age_ = 19.80 years; 30 females) participated for course credit. None of the participants in the current experiment participated in the previous experiment. Two participants from the current sample were removed from the results reported below because of a failure to fixate on the images for at least half of the experiment. In this experiment, participants also completed the trypophobia questionnaire designed by Le, Cole & Wilkins (2015). The questionnaire was completed after participants viewed all of the images during which their pupillary responses were recorded. Participants’ scores on the trypophobia questionnaire (*M* = 25.5, *Mdn* = 20, range = 17–83) fell within the normal range of the population who experience aversion to the images of holes but who may not self-identify as trypophobes (Le, Cole & Wilkins, 2015).

#### Stimuli and procedure

Stimulus presentation consisted of 60 images (512 × 512 px; 13.5°× 13.5°): 20 images of holes, 20 images of threatening animals (i.e., spiders and snakes), and 20 non-hole control images (see Fig. 1). The images of holes and threatening animals were identical to those used in Experiment 1. The non-hole control images consisted of individual objects or patterns with contrasting textures (e.g., checkerboard; see Fig. 1D). As in Experiment 1, all images were gray-scaled and equated for luminance using the SHINE toolbox for Matlab (mean luminance = 115.17; Mathworks; Willenbockel et al., 2010).

All aspects of image presentation were identical to Experiment 1. In particular, each trial began with the baseline phase (6 s) and was followed by the image phase (6 s). Images were presented in a randomized order. Any trials where participants did not fixate to the screen (during baseline or image phase) were excluded from statistical analyses (1.6% of trials). Participants were tested in a brightly lit room, seated in a chinrest 60 cm from the computer monitor. Eye gaze was calibrated using a 5-point calibration routine, and pupil area was recorded using an Eyelink-1000 plus eye-tracker (SR-Research) recording at 1,000 Hz.

In this experiment, participants also rated the images for level of arousal. Participants provided ratings of their subjective arousal to each image following the free viewing phase. Each image was presented onscreen (randomized order) with the question, “how does this image make you feel overall?” Participants responded on a 7-point scale ranging from -3 (“very negative”) to 3 (“very positive”). Arousal was calculated as the absolute value of the rating, regardless of valence (Bradley & Lang, 2007; but see, Kuppens et al., 2013, for alternative perspectives on measuring arousal).

We also collected ratings of fear and disgust in this experiment in an effort to corroborate the pupillary data. After rating images for arousal, participants rated each image (randomized order) on fear and disgust with the questions, “How fearful does this image make you feel?” and “How disgusted does this image make you feel?” on a 7-point scale ranging from 1 (“not at all”) to 7 (“extremely”).

#### Ethics

Participants provided written informed consent prior to participation. Experimental procedures were approved by the IRB at Emory University and performed in accordance with IRB guidelines (IRB protocol #003388).

### Results

As discussed above, the relatively greater pupillary constriction to images of holes could reflect high contrast, HSF information in these images. To address this possibility, we included control images with similar repetitive features and we then conducted an analysis of spatial frequency on the three image categories (holes, threat, and control) to ensure that the images were comparable. Images were analyzed for spatial frequency at five contrast energy levels (10%, 30%, 50%, 70%, and 90%; Bainbridge & Oliva, 2015). A Pearson correlation analysis revealed a negative relation between spatial frequency and pupil size, *rs*(58) =  − 0.415 to −0.616 (across energy levels), such that images with higher spatial frequencies elicited a smaller pupil, consistent with the pupil grating response (Barbur & Thomson, 1987; Cocker et al., 1994). Next, we compared stimulus categories across contrast energy levels with a 5 × 3 repeated-measures ANOVA with contrast energy (10%, 30%, 50%, 70%, and 90%) as the within-subjects factor and stimulus type (holes, threat, and control) as the between-subjects factor. This analysis revealed a main effect of contrast energy, *F*(4, 228) = 217.220, *p* < 0.001, ${\mathrm{\eta }}_{p}^{2}=0.792$, such that spatial frequency increased with contrast energy, as expected for this image set. But, critically, there was no main effect of stimulus type (*p* = 0.213, ${\mathrm{\eta }}_{p}^{2}=0.053$), nor a contrast energy by stimulus type interaction (*p* = 0.189, ${\mathrm{\eta }}_{p}^{2}=0.047$), demonstrating that spatial frequency was comparable across the image categories. However, because this analysis relies on the interpretation of a null effect, we also computed the expected power of this image set (Cohen, 1992), as well as the Bayes factor (BF_10_; Rouder et al., 2012) for the contrast energy by stimulus type interaction. These analyses revealed that with 60 images we had 99% power (1-β) to detect a medium-sized effect, and that the posterior distribution was in favor of the null hypothesis, BF_10_ = 0.220. Thus, although a pupil grating response was evident in our data, these analyses suggest that spatial frequency was comparable across the image categories at all contrast energy levels.

**Figure 3:**
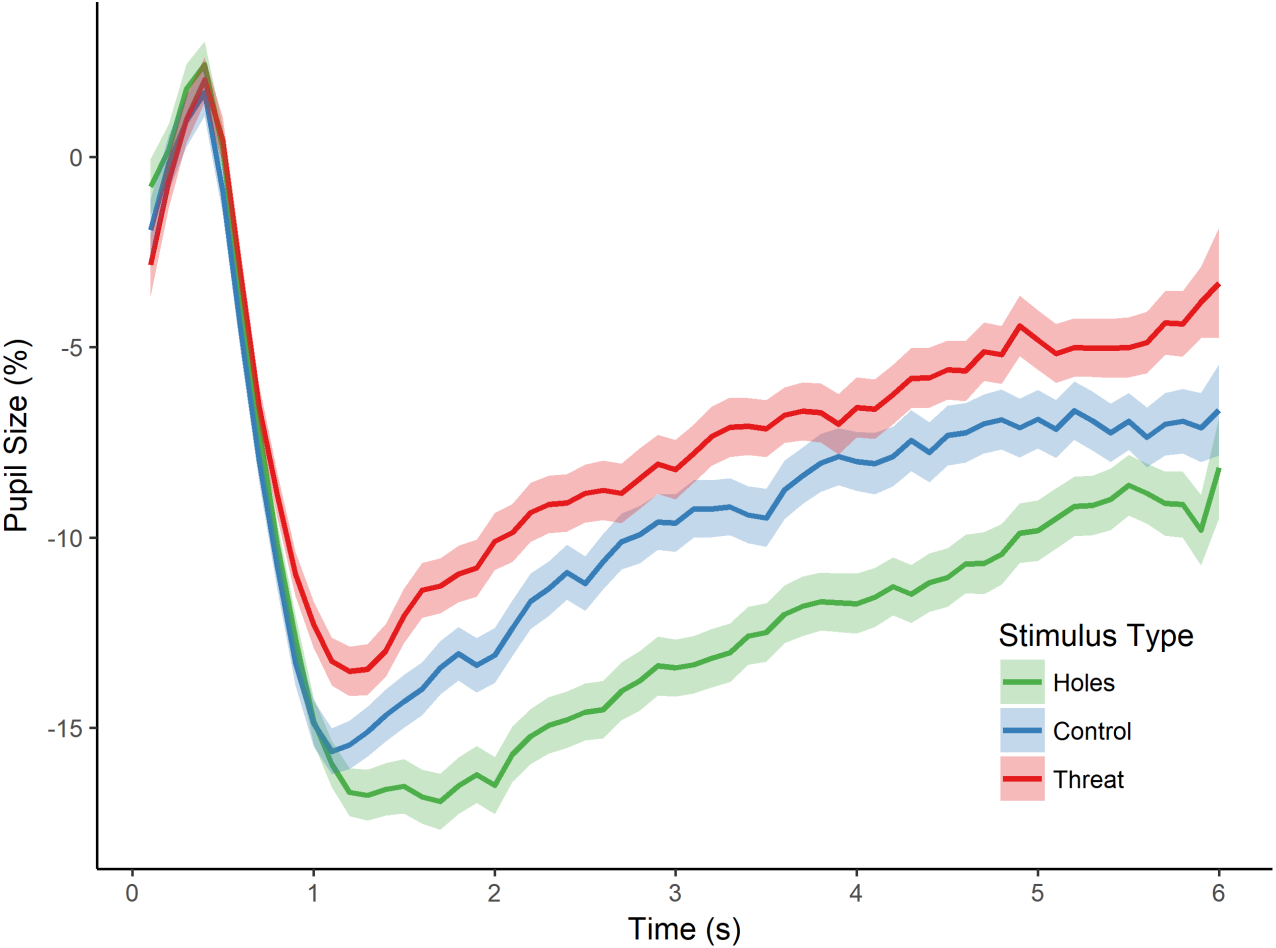
Pupillary waveforms across time for each stimulus type in Experiment 2. The *x*-axis reflects trial time in seconds (s) and the *y*-axis reflects the percentage of pupil-size change from baseline, such that greater percent change corresponds to a smaller pupil size. Shaded colors represent SEM.

As in the previous experiment, we then tested for an effect of stimulus type (i.e., holes, threat, and control) on pupillary size by conducting a repeated measures ANOVA with the dependent variable of percent pupil-size change. There was a significant main effect of stimulus type, *F*(2, 82) = 15.469, *p* < 0.001, ${\mathrm{\eta }}_{p}^{2}=0.274$ (see Fig. 3). Post-hoc tests (Holm-Bonferroni corrected) revealed that pupil-size change was, again, greater for holes than threatening images (*p* < 0.001), replicating the effect of Experiment 1. Moreover, and crucially, pupil-size change was also greater for holes than the control images (*p* < 0.001), which, in this experiment, consisted of high contrast repetitive patterns similar to holes, and which we confirmed were comparable in spatial frequency. Finally, there was a non-significant trend in the expected direction for the difference between threatening images and the control images (*p* = 0.085), with greater pupil dilation to spiders and snakes than control images. These findings replicate those of Experiment 1 by demonstrating a dissociation between pupillary responses to holes compared with threatening animals. They also demonstrate that the pupillary response to holes is not identical to that of other high-contrast, repetitive stimuli, thereby providing additional support for a parasympathetic response to holes and consistent with our hypothesis that holes elicit a disgust reaction.

However, an alternative possibility for these findings is that the images differed in arousal level. To address this possibility, we first conducted a one-way ANOVA on stimulus type (holes, threat, and control), which yielded a significant main effect of stimulus type on participants’ arousal ratings, *F*(2, 57) = 162.780, *p* < 0.001, ${\mathrm{\eta }}_{p}^{2}=0.851$. Post-hoc tests (Holm-Bonferroni corrected) revealed significant differences for all pairwise comparisons (*ps* < 0.001). Specifically, control images induced the least arousal, followed by holes, followed by the threatening category (see Table 1). Given these differences in arousal level, we conducted an additional analysis of stimulus type (holes, threat, and control) on pupil-size change while controlling for the effects of arousal. Participants’ pupil measurements for each image were regressed on arousal level, and a repeated-measures ANOVA was conducted on the residual values. This analysis yielded a significant main effect of stimulus type, *F*(2, 82) = 13.373, *p* < 0.001, ${\mathrm{\eta }}_{p}^{2}=0.246$. Post-hoc tests (Holm-Bonferroni corrected) revealed significant differences between holes and threatening images (*p* < 0.001), as well as between holes and controls (*p* < 0.001; no difference between threatening images and controls, *p* = 0.663), generally consistent with the analyses above. Taken together, the results from this experiment suggest that greater pupil constriction is specific to images of holes and not more generally applicable to non-hole repetitive images. Moreover, these results demonstrate that pupillary constriction to holes cannot be accounted for by differences in arousal level.

**Table 1:** Mean ratings of arousal, fear, and disgust for each stimulus category. Standard deviations in parentheses.

Stimulus type	Rating		
	Arousal	Fear	Disgust
Holes	0.91 (0.96)	1.94 (1.53)	2.47 (1.85)
Control	0.42 (0.79)	1.11 (0.50)	1.09 (0.44)
Threat	1.51 (1.06)	3.58 (2.19)	3.57 (2.24)

Finally, we examined participants’ fear and disgust ratings for each image (see Table 1), which converged with their pupillary responses. We conducted a 2 × 3 repeated measures ANOVA with emotion rating (fear and disgust) as the within-subjects factor and stimulus type (holes, threat, and control) as the between-subjects factor. This analysis revealed a main effect of emotion rating, *F*(1, 57) = 4.362, *p* < 0.001, ${\mathrm{\eta }}_{p}^{2}=0.415$, and stimulus type, *F*(2, 57) = 316.765, *p* < 0.001, ${\mathrm{\eta }}_{p}^{2}=0.917$, as well as an emotion rating by stimulus type interaction, *F*(2, 57) = 48.386, *p* < 0.001, ${\mathrm{\eta }}_{p}^{2}=0.629$. Post-hoc analyses (Holm-Bonferroni corrected) of these ratings revealed that images of holes were rated as more disgusting than fearful, (*p* < 0.001), as well as more disgusting than control images (*p* < 0.001). Critically, images of snakes and spiders were rated as more fear-inducing than both images of holes (*p* < 0.001) and control images (*p* < 0.001), but were rated as significantly more disgusting than images of holes, (*p* < 0.001; no difference between disgust and fear ratings for threatening images, *p* = 0.876). Taken together, participants’ ratings corroborate our interpretation of participants’ pupillary responses, as well as the anecdotal reports of self-described trypophobes, namely that the response to these images reflects disgust, not fear. The finding that images of snakes and spiders were rated as both fearful and disgusting relative to the other image categories was unexpected, but likely reflects either relatively greater non-specific arousal to the threatening animals (i.e., snakes and spiders were rated as more arousing than both holes and control images), or a combination of fear and disgust responses to threatening animals (see ‘General Discussion’).

### Discussion

In Experiment 2, we replicated the dissociation in participants’ pupillary responses to images of holes and threatening animals (snakes and spiders) reported in our first experiment. Moreover, we extended this finding by demonstrating greater pupil-size changes to holes than non-hole repetitive stimuli. Participants’ pupillary responses to holes were consistent with greater constriction compared with threatening animals and non-hole control images throughout the interest period. These findings rule out alternative explanations of pupillary constriction to images of holes. In particular, our findings cannot be accounted for by the pupil grating response since holes and control images elicited differential pupillary responses, despite comparable visual spectral properties. However, because this finding relies on a null spatial frequency difference between stimulus categories, future research would do well to ensure that pupillary constriction to trypophobic images occurs independently of spectral properties, or to assess the unique effects of spatial frequency and disgust on the pupillary response to holes. Importantly, though, our findings could not be accounted for by differences in arousal level since participants’ differential pupil responses to holes held when accounting for their subjective ratings of arousal to the images. Finally, participants’ explicit ratings provided confirmation that participants regarded images of holes as more disgusting than fearful. Taken together, we suggest that the current findings provide evidence for an emotional response to images of holes that reflects parasympathetic activation and, thus, most likely captures a disgust reaction.

## General Discussion

Across two experiments, we found a dissociation between images of holes, known to elicit trypophobia, and images of threatening animals (snakes and spiders). In particular, we found that holes, in contrast to the threatening animals and other control images, elicited larger pupil-size changes following baseline, a pattern of responding consistent with greater pupillary constriction during viewing. This pattern of pupillary responding suggests involvement of the parasympathetic nervous system while viewing images of holes, which we have argued is consistent with a disgust, not a fear, response.

Studies using vision science techniques (Cole & Wilkins, 2013; Le, Cole & Wilkins, 2015) and recent correlational research (Imaizumi et al., 2016a) suggest that trypophobia is largely rooted in the visual spectral properties of images. Moreover, it has been argued that general visual discomfort is associated with such spectral properties (Fernandez & Wilkins, 2008; O’Hare & Hibbard, 2011; Penacchio & Wilkins, 2015; Wilkins, 1995). Our research does not argue against this perspective. Indeed, an aversive reaction to images of holes and threatening animals such as snakes and spiders may stem from common visual properties. However, our work provides an important qualification on extant research by suggesting that these visual properties alone may not determine the specific emotional reaction experienced by the viewer. Indeed, we argue that although a general aversion may be rooted in visual spectral properties, the specific emotional reaction is determined by the content of the image. In other words, the emotional reaction may depend on an additional, top-down, cognitive appraisal. Consistent with this possibility is the acknowledgement from the original work of Cole & Wilkins (2013) in which it was stated that a trypophobic reaction is more likely for holes that appear on human skin as opposed to holes on inanimate objects (see also Kupfer & Le, 2017). If the aversion to these images were strictly due to the visual spectral properties of the image, this type of modulation would not be predicted. Although images of holes and those of snakes and spiders share a critical visual-spectral profile, individuals nevertheless show a dissociation in their pupillary responses, consistent with a distinction between physiological responses to disgusting and fearful stimuli.

Using electrodermal activity (EDA) and heartrate (BPM: heart-Beats Per Minute), Pipitone, Gallegos & Walters (2017) recently examined participants’ physiological responses to images of holes (similar to those used in the present study). They found that images of holes elicited higher levels of EDA relative to control images, which is consistent with a sympathetic nervous system response, but in apparent contradiction to our findings, which suggests a parasympathetic response to holes. It should be noted, however, that BPM did not converge with the EDA result. Pipitone, Gallegos & Walters (2017) found that BPM did not dissociate trypophobic and control images. In fact, BPM was lower, though not statistically, to holes than control images (*p* = 0.1, *d* =  − 0.28), which would suggest parasympathetic activation. Moreover, because ratings of arousal were not taken into consideration, it is unclear whether a sympathetic nervous system response could be accounted for by differences in arousal to the trypophobic and control images. It thus remains an important question for future research to determine whether different measures–pupillometry, electrodermal activity and heartrate–provide converging support for a common physiological response to images of holes, suggestive, as proposed here, of a disgust emotional response (Mauss & Robinson, 2009).

The current findings are consistent with evolutionary perspectives that hold that fear has roots in danger avoidance and predator–prey interactions. Disgust, however, may instead allow for the avoidance of sources of disease such as rotten food or the visibly sick (Woody & Teachman, 2000). It has been suggested that a trypophobic reaction may be an extension of an intrinsic disgust for decomposing items, sores and scars, which would aid in the avoidance of contaminated stimuli specifically and disease more generally (Cole & Wilkins, 2013; Deacon & Olatunji, 2007; Rozin & Fallon, 1987; Skaggs, 2014). Holes, but not spiders and snakes, or other repetitive patterns, may have come to be associated with disease transmission (Rozin & Fallon, 1987), either over the course of evolution or learned during ontogenetic development (Can, Zhuoran & Zheng, 2017; Kupfer & Le, 2017). The result of this association may be a corresponding withdrawal response controlled by the parasympathetic nervous system.

Nevertheless, one question left unanswered by our data concerns the muted pupillary dilation to snakes and spiders. In neither experiment did we find definitive evidence that changes in pupil size were consistent with larger dilation for snakes and spiders in comparison to control images (the effect in Experiment 2 did not reach statistical significance), which one might have predicted if threatening animals elicit a fear response. One possibility is that the participants in this study were simply not especially fearful of these stimuli. Another possibility is that the response to snakes and spiders reflects a combination of fear and disgust such that the additional component of disgust tempers the pupillary dilation. Indeed, our own ratings and previous research suggests that spider phobics report both fear and disgust to spider photos, with a strong correlation between the two emotions (Thorpe & Salkovskis, 1998). It is therefore possible that the contrasting influence of both fear and disgust could have muted pupillary dilation in our participants. Future research would do well to investigate pupillary responses to stimuli that elicit a fear or disgust response exclusively.

A possible concern about the present work is that participants may not have been trypophobes. However, we would argue that the use of a normative sample of participants should be considered a strength. That we found a pupillary dissociation between holes and images of spiders and snakes, even when the accompanying emotions were likely not at the extreme is important because it allows for a crucial baseline by which to subsequently compare phobic individuals. In Experiment 2 of the present study, some individuals would qualify as trypophobes based on their scores on the trypophobia questionnaire (values >31; Le, Cole & Wilkins, 2015). However, given the relatively small number of participants who met this criterion (*N* = 9), we did not conduct separate analyses on these participants. One possibility is that the results reported in the present study will replicate in a large sample of trypophobes and that the dissociation will be even more pronounced. Indeed, one could straightforwardly predict stronger effects in these individuals. However, another possibility is that the dissociation might be less pronounced in the trypophobes. Perhaps trypophobes will experience some combination of disgust and fear, as has been found for spider phobics (Thorpe & Salkovskis, 1998). The fact that spider phobics show a correlation between these emotions when viewing images of spiders would be consistent with such a possibility. Future research might consider making greater efforts to include both types of populations (phobics and non-phobics) to understand the full spectrum of fear and disgust.

## Conclusion

Despite trypophobes describing their aversion to holes as “disgusting,” the emotional response to images of holes has been characterized as “fearful” because of how similar their visual spectral properties are to images of threatening animals. Here we used pupillometry to investigate the physiological underpinnings of the aversion to holes in order to determine whether the specific emotional response reflects fear or disgust. Across two experiments, we found greater pupillary constriction to holes than to snakes and spiders, as well as different types of neutral images. Importantly, this effect could not be explained by arousal or low-level visual properties (though more research would be useful to ensure that spatial frequency alone does not account for pupillary responses). These findings are consistent with involvement of the parasympathetic nervous system when viewing images of holes and is suggestive of a disgust, not fear, response to these images. These data suggest that although a general aversion may be rooted in common visual spectral properties, the specific emotional response may reflect cognitive appraisal of image content.

##  Supplemental Information

10.7717/peerj.4185/supp-1 Data S1 Aversion to holes—raw data for all experiments

## Additional Information and Declarations

### Data Availability

The following information was supplied regarding data availability: The raw data is provided as Data S1.
